# Glutamine induces heat-shock protein and protects against *Escherichia coli *lipopolysaccharide-induced vascular hyporeactivity in rats

**DOI:** 10.1186/cc5717

**Published:** 2007-03-09

**Authors:** Liang Jing, Qiong Wu, Fuzhou Wang

**Affiliations:** 1 Department of Anesthesiology, Zhongda Hospital and School of Clinical Medicine, Southeast University, 87# Ding Jia Qiao Rd., Nanjing, Jiangsu, China

## Abstract

**Introduction:**

Vascular hyporeactivity is an important problem associated with sepsis. Although the mechanism involves inflammatory pathway activation, specific therapeutic approaches have not been defined. Glutamine (Gln) has been shown to provide some anti-inflammatory effects and improve outcomes in sepsis. Here, we tested the hypothesis that Gln could reduce *Escherichia coli *lipopolysaccharide (LPS)-induced vascular hyporeactivity and evaluated the role of heat-shock protein 70 (HSP70) induction in this process.

**Methods:**

Twenty-four male Sprague-Dawley rats were divided into control, LPS shock, and alanyl-Gln dipeptide+LPS shock (Ala-Gln+LPS) groups. Six hours after administration of LPS, phenylephrine (PE) (0.5 to approximately 2.5 μg/kg) was applied intravenously to all groups, and the percentage increase in mean arterial pressure (MAP) was detected in the respective groups. The concentration-response curve of PE was obtained in tension experiments, and the average values of PE maximum efficacy (E_max_) and median effective dose (EC_50_) were calculated. The plasma concentrations of malondialdehyde (MDA), tumor necrosis factor-alpha (TNF-α), and interleukin-6 (IL-6) were detected in all groups. The expressions of HSP70 from heart, liver, lung, and aorta were also assayed in all groups.

**Results:**

The maximal percentage increase in MAP induced by PE was significantly reduced to 12.7% in the LPS shock group (*P *< 0.05) and was restored to 15.6% in the Ala-Gln+LPS group (*P *< 0.05), whereas the control group was 24.7%. The average values of PE E_max _and EC_50 _were significantly impaired in the LPS shock group (*P *< 0.05) but partially restored in the Ala-Gln+LPS group (*P *< 0.05). The expressions of HSP70 from the heart, aorta, lung, and liver were much higher in the Ala-Gln+LPS group than those in the LPS shock group (*P *< 0.05). The plasma concentrations of TNF-α, IL-6, and MDA were much lower in the Ala-Gln+LPS group than those in the LPS shock group.

**Conclusion:**

Gln effectively improves vascular reactivity by inducing the expression of HSP70, reducing inflammatory cytokine release and peroxide biosynthesis in LPS shock rats. These results suggest that Gln has a potentially beneficial therapeutic effect for septic shock patients.

## Introduction

Septic shock is a complex pathophysiological state, and despite considerable therapeutic advances, it remains a major therapeutic challenge with a high incidence of mortality [1]. Vascular hyporeactivity to catecholamine vasoconstrictors is a characteristic feature of septic shock, plays a key role in this pathological process, and results in arterial hypotension, multiple organ dysfunction, and death. The underlying mechanism of impaired vasopressor responsiveness in septic shock is not completely understood but likely involves the activation of inflammatory pathways [2].

The therapeutic approaches for the treatment of vascular hyporeactivity in septic shock have included using high-dose vasoactive agents, nitric oxide synthases inhibitors [3], guanylate cyclase inhibitor [4], low-dose corticosteroids [5], and antioxidant therapy [6]. These have been experimentally used in clinical and animal studies, but their value in therapeutics is not proven. Thus, the precise mechanisms of cardiovascular dysfunction during sepsis warrant further study and the new therapeutic approaches should be explored.

Heat-shock proteins (HSPs) are self-protective proteins that maintain cell homeostasis against various forms of stress as an adaptive response [7]. These proteins are induced by a wide variety of stressors and have broad cytoprotective functions. The 70-kDa family of HSP (HSP70), in particular, plays a vital role in cellular protection and has been detected in various tissues subject to stress [8,9]. Heat stress, gene transfer, and some small-molecule agents have been reported to induce HSP70 expression [10-12], but the potential clinical value of these approaches has not been defined.

Glutamine (Gln), a non-essential amino acid, has been demonstrated to attenuate pro-inflammatory cytokine release [13] and lung metabolic dysfunction in animal models of endotoxin shock through enhanced HSP expression [14]. No previous studies have evaluated the impact of Gln administration on sepsis-related vascular hyporeactivity. In this study, we examined the hypothesis that pretreatment of Gln could induce HSP70 expression and improve vascular reactivity in a relevant rat model of lipopolysaccharide (LPS)-induced sepsis.

## Materials and methods

### Animals

The study was approved by the Ethical Committee of Animal Research at the College of Medicine, Southeast University, Nanjing, China. Twenty-four healthy male Sprague-Dawley rats weighing 250 to approximately 300 g were randomly divided into three groups: a control group, which received an intravenous infusion of 5 to 7 ml of lactated Ringer's solution (LR) (*n *= 8); an LPS shock group, which received an intravenous infusion of 5 to 7 ml of LR until one hour before intravenous administration of LPS (Sigma-Aldrich, St. Louis, MO, USA) 10 mg/kg (*n *= 8); and an alanyl-Gln dipeptide+LPS shock (Ala-Gln+LPS) group, which received an intravenous infusion of 5 to 7 ml of 4% Ala-Gln until one hour before intravenous administration of LPS (*n *= 8). All fluids were infused by micropump at a rate of 5 to 7 ml/hour.

### Glutamine administration

Gln was administered as 20% Ala-Gln (Fresenius Kabi Austria GmbH, Graz, Austria), which was diluted into 4% solution with LR for intravenous infusion because Ala-Gln must be diluted five times for intravenous administration in clinical application. Five to seven milliliters of 4% Ala-Gln was administered to yield 0.75 g/kg per dose of Gln. Ala-Gln solution or LR vehicle was administered via femoral vein injection.

### Measurement of mean arterial pressure

All rats were anesthetized with sodium pentobarbital (40 mg/kg intraperitoneally), and a supplemental dose (20 mg/kg) was added if necessary. The rats were allowed to keep breathing spontaneously. One catheter was placed in the femoral artery and connected to the pressure transducers for recording mean arterial pressure (MAP), and another one was placed in the femoral vein as a route for drug administration. MAP values of the rats were decreased after administration of LPS in the LPS group and the Ala-Gln+LPS group, and an MAP decrease of 25% to 30% of baseline level was regarded as endotoxin shock [15]. Six hours after administration of LPS, phenylephrine (PE) (Shanghai Harvest Pharmaceutical Co., Ltd., Shanghai, China) in doses of 0.5, 1.0, 2.0, and 2.5 μg/kg was applied every 20 minutes via the femoral vein; the percentage increase in MAP was recorded in each group.

### Isolated vascular function

All rats were anesthetized by sodium pentobarbital and killed by decapitation after levels of MAP were measured. The thoracic aorta was rapidly isolated and prepared with the endothelium intact. Vascular segments (3 to 4 mm) were suspended by stainless steel hooks in 10-ml tissue baths containing Krebs buffer at 37°C, oxygenated by constant bubbling of a 95%/5% O_2_/CO_2 _mixture, and incubated for 90 minutes. Tension data were collected with the Biologic Signal Collecting System (Nanjing Medical University, Nanjing, China). After five washes, concentration effect data were obtained by cumulative addition of PE (1 × 10^-9 ^to 1 × 10^-4 ^M; Sigma-Aldrich).

### HSP70 protein expression detection with Western blotting analysis

Six hours after injection of LPS, the heart, liver, lung, and aorta were harvested, immediately frozen in liquid nitrogen, and stored at -80°C until analysis. Tissues were homogenized in buffer (10 mM Tris, 5 mM EDTA [ethylenediaminetetraacetic acid], 2% Triton X-100, 0.2 mM Na_3_VO_4_, 1 mM phenylmethylsulfonyl fluoride, and 10 μg/ml leupeptin and aprotinin) and mechanically disrupted. Samples were analyzed by SDS-PAGE by means of a transfer buffer (25 mM/l Tris, 192 mM/l glycine, and 20% methanol) in a wet-transfer apparatus. Blots were blocked with 5% non-fat dry milk in phosphate-buffered saline with 0.1% Tweens-20 and then incubated with mouse anti-rat monoclonal HSP70 antibody (Sigma-Aldrich). After repeated washing, rabbit anti-mouse secondary antibody (horseradish peroxidase-conjugated) incubation was performed, developed with a chemiluminescence system, and followed with film exposure and relative intensity analysis.

### Detection of plasma tumor necrosis factor-alpha, interleukin-6, and malondialdehyde

The arterial blood sample (1.5 ml) was collected from all groups at 90 minutes (for tumor necrosis factor-alpha [TNF-α] detection, plasma peak was thought to be achieved within two hours after LPS injection [16]) and six hours (for plasma interleukin-6 [IL-6] and malondialdehyde [MDA] detection) after administration of LPS. Blood was then centrifuged for eight minutes at 3,500 *g *and 4°C, and the supernatant was collected. The plasma IL-6 and TNF-α were analyzed using enzyme-linked immunosorbent assay kits (Shanghai Hua Sen Science & Technology CO. Ltd., Shanghai, China). Results were then obtained using a microplate reader (Model 680; Bio-Rad Laboratories, Inc, Tokyo, Japan). The content of plasma MDA levels was measured by means of a thibabituric acid reaction.

### Statistical analysis

Concentration-response data were fitted to a sigmoidal maximum efficacy (E_max_) model by means of GraphPad Prism software (GraphPad Software, Inc., San Diego, CA, USA). The values of PE E_max _and median effective dose (EC_50_) were determined for each treatment group. Data are presented as mean ± standard deviation. Statistical analyses were performed using one-way analyses of variance. All analysis was performed using the Statistical Software Package (SPSS version 11.5; SPSS Inc., Chicago, IL, USA). Statistical significance was assigned at a *P *value of less than 0.05.

## Results

### Changes in mean arterial pressure in response to phenylephrine

**Figure 1:**
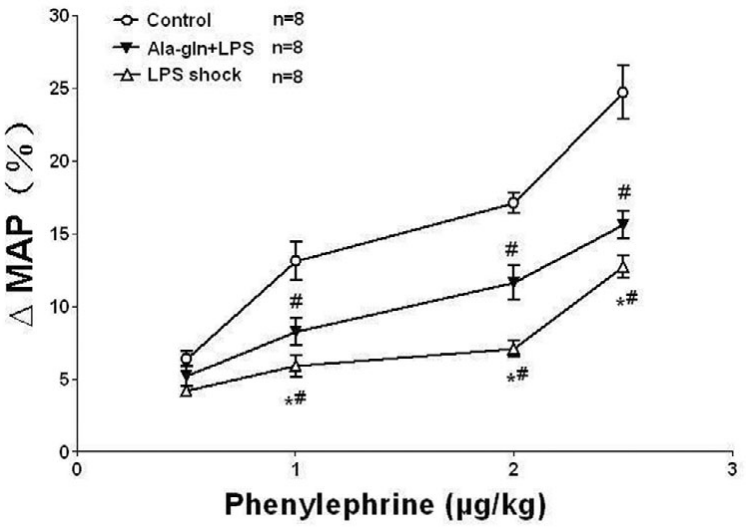
The percentage increase in mean arterial pressure (MAP) induced by phenylephrine in different groups of rats. The maximal percentage increase in MAP significantly decreased to 12.7% in the LPS shock group (*P *< 0.05) and was restored to 15.6% in the Ala-Gln+LPS group, whereas the maximum percentage increase in the control group was 24.7% (*n *= 8, mean ± standard deviation). *P < 0.05 versus the Ala-Gln+LPS group; ^#^*P *< 0.05 versus the control group. Ala-Gln+LPS, alanyl-glutamine dipeptide + lipopolysaccharide shock; LPS shock, lipopolysaccharide shock.

There were no significant differences in baseline levels of MAP among the groups, but MAP decreased to 75% to 70% of the baseline level six hours after administration of LPS. PE produced a dose-dependent increase in MAP in all groups, but the maximal percentage increase in MAP significantly decreased to 12.7% in the LPS shock group (*P *< 0.05) and was restored to 15.6% in the Ala-Gln+LPS group whereas the maximal percentage increase in the control group was 24.7% (*P *< 0.05) (Figure 1).

### Isolated vascular response to phenylephrine

**Figure 2:**
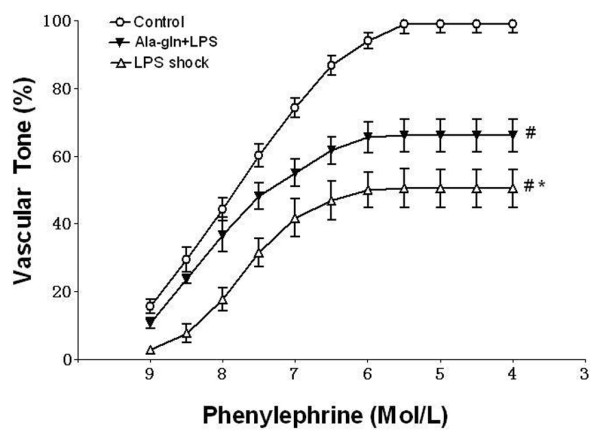
The concentration-response curves of phenylephrine (PE) in aortic rings from different groups of rats (mean ± standard deviation). PE maximum efficacy (E_max_) significantly decreased to 51% in the LPS shock group and was restored to 68% in the Ala-Gln+LPS group (*P *< 0.05), whereas PE E_max _in the control group was taken as 100%. *P < 0.05 versus the Ala-Gln+LPS group. #*P *< 0.05 versus the control group. Ala-Gln+LPS, alanyl-glutamine dipeptide + lipopolysaccharide shock; LPS shock, lipopolysaccharide shock.

In the vascular tension experiments, each tissue developed tension to PE in a concentration-dependent way. The average values of PE EC_50 _and E_max _in the control group were 8.55 ± 0.08 nmol/l and 1.86 ± 0.05 g, respectively (Table 1). PE E_max _significantly decreased to 51% (0.95 ± 0.01 g) in the LPS shock group and was restored to 68% (1.27 ± 0.03 g) in the Ala-Gln+LPS group, whereas PE E_max _in the control group was taken as 100% (*P *< 0.05) (Figure 2, Table 1). Likewise, fitted PE EC_50 _significantly increased to 13.49 ± 0.06 nmol/l in the LPS shock group and was reversed to 10.15 ± 0.04 nmol/l in the Ala-Gln+LPS group (*P *< 0.05) (Table 1).

**Table 1:** The values of PE E_max _and EC_50 _on aortic rings in the different groups of rats

Group	E_max _(g)	EC_50 _(nmol/l)
Control	1.86 ± 0.04	8.55 ± 0.08
LPS shock	0.95 ± 0.01^a^	13.49 ± 0.06^a^
Ala-Gln+LPS	1.27 ± 0.02^a,b^	10.15 ± 0.04^a,b^

Data are shown as mean ± standard deviation (*n *= 8 in each group). ^a^*P *< 0.05 versus the control group; ^b^*P *< 0.05 versus the LPS shock group. Ala-Gln+LPS, alanyl-glutamine dipeptide + lipopolysaccharide shock; E_max_, maximum efficacy; EC_50_, median effective dose; LPS, lipopolysaccharide.

### Glutamine enhances HSP70 expression

**Figure 3:**
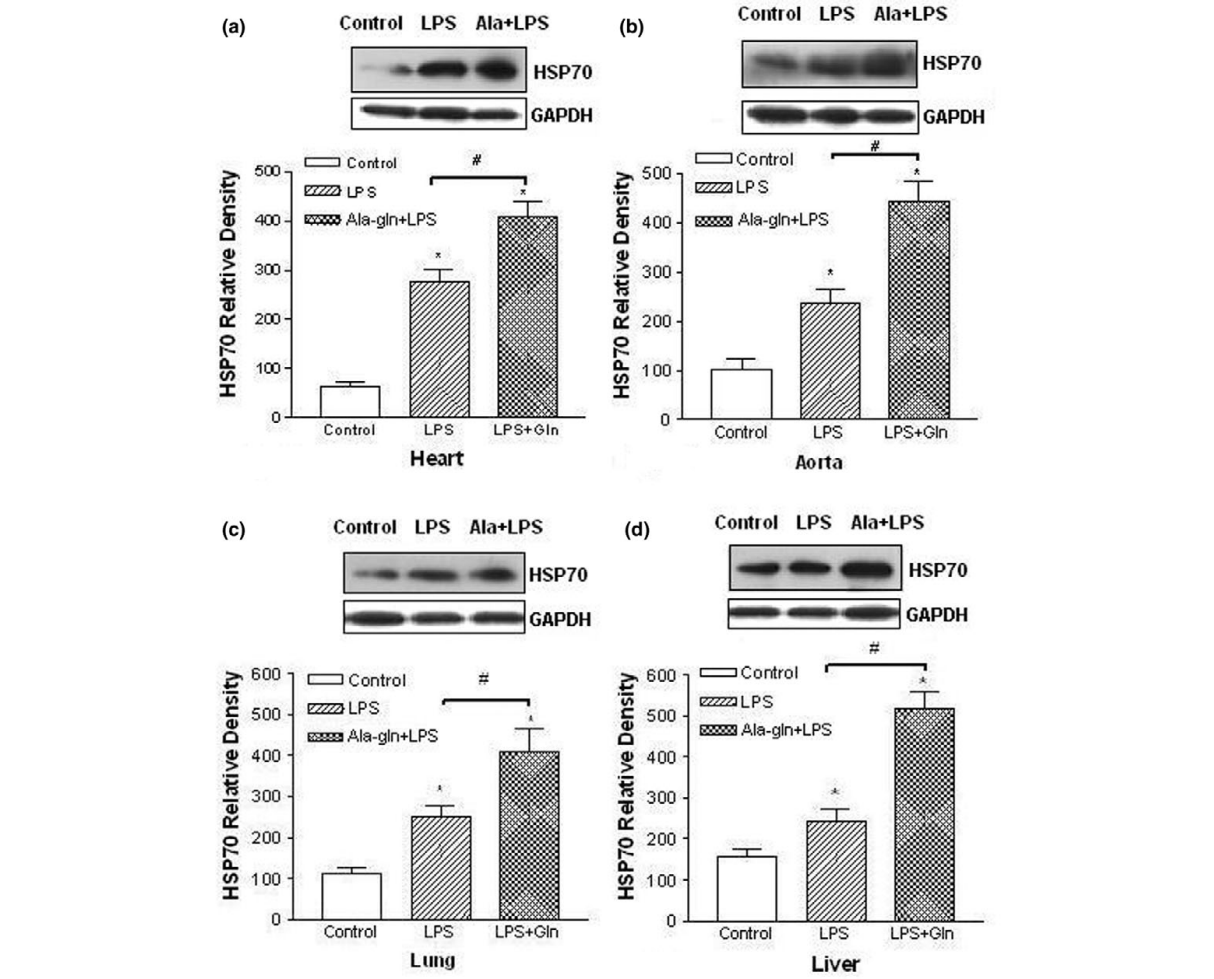
Effects of alanyl-glutamine on heat-shock protein 70 (HSP70) expression in heart, aorta, lung, and liver in endotoxin rats. HSP70 expressions were analyzed by Western blotting analysis. Relative density refers to the ratio of HSP70 to GAPDH. The expression of HSP70 was significantly increased after lipopolysaccharide (LPS) injection compared with the control group in heart **(a)**, aorta **(b)**, lung **(c)**, and liver **(d) **tissue. (*P < 0.05; *n *= 5). The expressions of HSP70 were much higher than those in the LPS shock group from four tissues in the Ala-Gln+LPS group (#*P *< 0.05; *n *= 5). Ala-Gln+LPS, alanyl-glutamine dipeptide + lipopolysaccharide shock; Ala+LPS, alanyl-glutamine dipeptide + lipopolysaccharide shock; GAPDH, glyceraldehyde-3-phosphate dehydrogenase; LPS+Gln, alanyl-glutamine dipeptide + lipopolysaccharide shock.

The analyses from Western blotting showed that HSP70 expressions in heart tissue (Figure 3a), aorta tissue (Figure 3b), lung tissue (Figure 3c), and liver tissue (Figure 3d) were weak in the control group but markedly stronger in the LPS shock group (*P *< 0.05). The expressions of HSP70 were much higher than those in the LPS shock group from four tissues in the Ala-Gln+LPS group (*P *< 0.05) (Figure 3a–d).

### Glutamine decreases plasma concentrations of TNF-α, IL-6, and MDA

The plasma TNF-α, IL-6, and MDA levels were low in the control group (49.7 ± 12.2 pg/ml, 23.5 ± 9.2 pg/ml, and 4.66 ± 0.55 mol/ml, respectively). They significantly increased in the LPS shock group (293.1 ± 52.2 pg/ml, 296.2 ± 60.2 pg/ml, and 9.71 ± 0.87 mol/ml, respectively; *P *< 0.01) (Table 2). Pretreatment with Ala-Gln significantly decreased plasma concentrations of TNF-α, IL-6, and MDA compared with the LPS shock group (131.8 ± 27.7 pg/ml, 204.1 ± 42.2 pg/ml, and 5.89 ± 0.58 mol/ml, respectively; *P *< 0.05) (Table 2).

**Table 2:** Plasma concentrations of TNF-α, IL-6, and MDA in the different groups of rats

Group	TNF-α (pg/ml)	IL-6 (pg/ml)	MDA (mol/ml)
Control	49.7 ± 12.2	23.5 ± 9.2	4.66 ± 0.55
LPS shock	293.1 ± 52.2^a^	296.2 ± 60.2^a^	9.71 ± 0.87^a^
Ala-Gln+LPS	131.8 ± 27.7^a,b^	204.1 ± 42.2^a,b^	5.89 ± 0.58^a,b^

Data are shown as mean ± standard deviation (*n *= 8 in each group). ^a^*P *< 0.05 versus the Ala-Gln+LPS group; ^b^*P *< 0.05 versus the control group. Ala-Gln+LPS, alanyl-glutamine dipeptide + lipopolysaccharide shock; IL-6, interleukin-6; LPS, lipopolysaccharide; MDA, malondialdehyde; TNF-α, tumor necrosis factor-alpha.

## Discussion

The results of this study demonstrate that pretreatment of Ala-Gln significantly improved vascular response to catecholamine vasoconstrictors and that the effect of Ala-Gln is associated with its capacity to induce HSP70 expression and attenuate release of pro-inflammatory cytokines and oxidizing species production after septic shock. HSP70 is the most important protein in HSP family to generate a protective effect against injuries in the presence of various stresses [17]. Previous studies in rat models of HSP induction to protect against septic shock have used sodium arsenite or heat as an inducer for the stress response. Sodium arsenite is known to be quite toxic; a previous experiment showed a 20% mortality rate from the arsenite alone [18]. HSP expression has also been induced by measures that increase core body temperature [19]. However, these measures are clinically impractical because they would be poorly tolerated by patients and would have detrimental effects on many cellular functions [20]. Gln may have therapeutic value in safely and effectively enhancing the expression of HSP and may increase the survival from septic shock [21]. Therefore, we chose Ala-Gln as an HSP expression inducer in this study.

In the present study, we designed pretreatment of 0.75 g/kg Gln to be one hour before LPS injection and performed a vascular functional test six hours after administration of LPS. The dose of Gln dipeptide used in this study was based on our previous data, which indicate that the maximal HSP70 mRNA expression in rats occurs at 6 to approximately 12 hours after an intravenous dose of 0.75 g/kg Gln. This dose has also been demonstrated to safely induce HSP70 expression in sepsis rats [22]. One limitation of this study seems to be that we chose the pretreatment of Ala-Gln rather than to treat at the onset or after a septic injury since the latter is more likely to get close to the clinical utility. This was done because from our previous experimental data, we know that the maximal HSP70 expression occurs at six hours after Ala-Gln is used. However, administration of LPS results in hypotension at the third to fourth hour in rats [23]. If Gln injection time is same with or post LPS application, maybe could not exactly evaluate the protective effect of Gln on vascular reactivity because Gln-induced maximal HSP expression dose not obtained.

A potential limitation of this study is that we chose to use a vehicle-based control rather than an iso-nitrogenous amino acid control. Previous studies have shown that alanine does not lead to significant enhancement of HSP70 either in animals [24] or patients [25], suggesting that the pharmacological effects we have observed are related to the Gln treatment.

Ala-Gln must be diluted five times for intravenous administration based on its description; therefore, there is an excessive amount of fluid infusion in the present study. This acute volume overloading results in slight increases in blood pressure and heart rate but does not lead to death in rats. The effects of volume overloading on the measurement of MAP should be considered. However, the volume of infusion in every administration was strictly controlled according to body weight to be the same in the three groups, and the MAP still decreased after administration of LPS. This indicates that, although volume overloading could temporarily change the hemodynamics in rats, the change of MAP could still be considered a sensitive parameter that reflected vascular response to agonists in the present study. Furthermore, a total of 3 ml of blood was drawn at 90 minutes and 6 hours before the start of the experiment and this may have partially reduced the influence of hypervolemia.

Endotoxin shock is characterized by a marked oxidant stress [26] and a rapid production of different cytokines [27]. Nuclear factor-kappa-B is a transcription factor that plays a central role in the modulation of the inflammatory and immune responses and induces the expression of many genes of inducible nitric oxide synthase, cytokine tissue factor, and adhesion molecules involved in the pathogenesis of endotoxin shock [28]. The results of our study showed that the levels of plasma TNF-α, IL-6, and MDA in the Ala-Gln+LPS group were lower than those in the LPS shock group, indicating that the inhibited pro-inflammatory cytokine release and peroxide production may also be attributed to the protective effects of Gln on LPS-induced vascular hyporeactivity. The mechanism involved may be that Gln inhibits the expression of the inflammatory cytokines directly [22] or that Gln-induced HSP70 expression further enhanced this effect [29].

The data provided in this study demonstrate two discrete mechanistic effects produced by Ala-Gln, namely the reduced LPS-induced cytokine presence in plasma and the increased HSP70 in multiple tissues. The actual upstream mechanisms responsible for these changes are not clear, but they may be separately regulated and influenced by Ala-Gln. We hypothesize that this dual effect of decreased cytokine-induced vascular injury/action and increased vascular cell survival may be critical for the improved outcomes we observed. Further studies to define the molecular pathways responsible for these discrete actions are clearly warranted, as is further investigation of Gln as a modulator of sepsis-related cardiovascular outcomes.

## Conclusion

Gln has been well demonstrated to protect against organ dysfunction in animal experiments and in critically ill patients by inducing HSP70 expression, attenuating sepsis-induced metabolic dysfunction, and reducing inflammatory cytokine release and peroxide production. Here, we further demonstrated that administration of a dose of 0.75 g/kg Gln could protect against vascular hyporeactivity in endotoxic shock rats. Thus, Ala-Gln could be used to induce the protective stress response and prevent end-organ injury and possibly decrease mortality from sepsis and improve outcomes in critically ill patients.

## Key messages

• Alanyl-glutamine improves vascular hyporeactivity in endotoxic shock rats.

• The protective role of alanyl-glutamine on vascular reactivity comes from inducing HSP70 expression and reducing inflammatory cytokine release and peroxide biosynthesis.

• These results suggest that alanyl-glutamine has potentially beneficial therapeutic effects in sepsis.

## Abbreviations

Ala-Gln+LPS = alanyl-glutamine dipeptide + lipopolysaccharide shock; EC_50 _= median effective dose; E_max _= maximum efficacy; Gln = glutamine; HSP = heat-shock protein; HSP70 = heat-shock protein 70; IL-6 = interleukin-6; LPS = lipopolysaccharide; LR = lactated Ringer's solution; MAP = mean arterial pressure; MDA = malondialdehyde; PE = phenylephrine; TNF-α = tumor necrosis factor-alpha.

## Competing interests

The authors declare that they have no competing interests.

## Authors' contributions

LJ carried out the design of the study, established the experimental setup, drafted the manuscript, and participated in part of the animal experiments. QW carried out the *in vivo *and *in vitro *animal experiments and blood analysis and performed the statistical analysis. FW carried out the HSP70 protein expression detection. All authors read and approved the final manuscript.
